# Multicore-based ferrofluids in zero field: initial magnetic susceptibility and self-assembly mechanisms

**DOI:** 10.1039/d3sm00440f

**Published:** 2023-06-07

**Authors:** Andrey A. Kuznetsov, Ekaterina V. Novak, Elena S. Pyanzina, Sofia S. Kantorovich

**Affiliations:** a Computational and Soft Matter Physics, University of Vienna Kollingasse 14-16 1090 Vienna Austria andrey.kuznetsov@univie.ac.at; b Institute of Natural Sciences and Mathematics, Ural Federal University Lenin av. 51 620000 Ekaterinburg Russia; c Research Platform MMM Mathematics-Magnetism-Material, University of Vienna Oskar-Morgenstern-Platz 1 1090 Vienna Austria

## Abstract

The necessity to improve magnetic building blocks in magnetic nano-structured soft materials stems from a fascinating potential these materials have in bio-medical applications and nanofluidics. Along with practical reasons, the interplay of magnetic and steric interactions on one hand, and entropy, on the other, makes magnetic soft matter fundamentally challenging. Recently, in order to tailor magnetic response of the magnetic particle suspensions, the idea arose to replace standard single-core nanoparticles with nano-sized clusters of single-domain nanoparticles (grains) rigidly bound together by solid polymer matrix – multicore magnetic nanoparticles (MMNPs). To pursue this idea, a profound understanding of the MMNP interactions and self-assembly is required. In this work we present a computational study of the MMNP suspensions and elucidate their self-assembly and magnetic susceptibility. We show that depending on the magnetic moment of individual grains the suspensions exhibit qualitatively distinct regimes. Firstly, if the grains are moderately interacting, they contribute to a significant decrease of the remanent magnetisation of MMNPs and as such to a decrease of the magnetic susceptibility, this way confirming previous findings. If the grains are strongly interacting, instead, they serve as anchor points and support formation of grain clusters that span through several MMNPs, leading to MMNP cluster formation and a drastic increase of the initial magnetic response. Both the topology of the clusters and their size distribution in MMNP suspensions is found to be notably different from those formed in conventional magnetic fluids or magnetorheological suspensions.

## Abstract 2

Magnetic multicore particles are not only possessing a high potential in biomedical applications, but are also exhibiting fundamentally different magnetic response and self-assembly if compared to their single-core counterparts.

## Graphical abstract

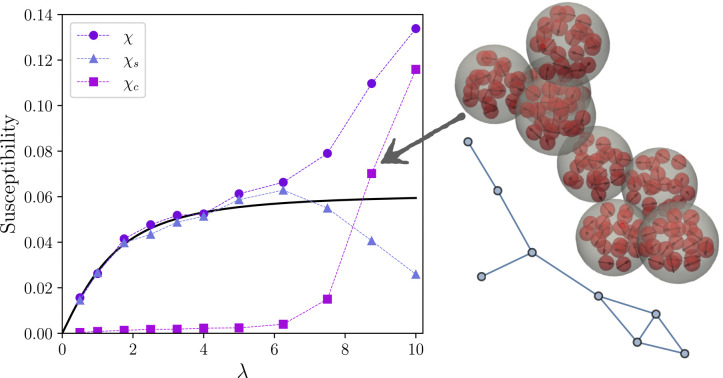

## 1 Introduction

Nowadays, the idea of using smart nano-textured materials that we can thoroughly control and manipulate with external stimuli forms the basics for novel medical applications. In particular, when it comes to magnetic fields that are not interfering with any biological or physiological processes,^1^ magnetic soft matter systems have gained a lot of attention for their potential in drug targeting^2–8^ and magnetic hyperthermia.^9–12^ The beginning of the magnetic soft matter field can be associated with the synthesis of ferrofluids back in the middle of the twentieth century.^13^ Today they are regarded as, probably, the simplest, albeit not fully understood and exploited, example of magneto-controllable material. Ferrofluids (or magnetic fluids) consist of two components: magnetic single-domain nanoparticles suspended in liquid magneto-passive carriers. It is the polydispersity of magnetic nanoparticles, peculiarities of their colloidal stabilisation and the resulting span of time-, length- and energy-scales that make ferrofluids difficult to manipulate in a highly controlled manner. In fact, any structures that nanoparticles form in ferrofluids are very sensitive to thermal noise and mechanical perturbations. Instead, it was recently suggested to replace single-domain magnetic particles by preassembled complex objects such as supramolecular polymer-like structures made of magnetic monomers,^14–24^ magnetic nano- and micro-gels,^4,25,26^ or magnetic multicore nanoparticles (MMNPs).^27–30^ The suspensions of the latter were recently even addressed as “*bio-ferrofluids*”.^31^ This name is not just metaphoric, as MMNPs already gave rise to innovations in drug delivery,^32,33^ magnetic particle imaging,^27,34^ magnetic hyperthermia cancer treatment^35–38^ and immunoassays.^39,40^ Along with biomedical applications MMNPs have been used in nanorheology.^41–46^ In all these works, one can think of a single MMNP as of a cluster of single-domain magnetic nanocrystals (below in this work addressed as “*grains*”) embedded in a polymer or other non-magnetic rigid matrix.^47,48^ While the grains typically have a characteristic linear size of the order of 10 nm, the size of MMNPs can range from tens to a few hundred nanometers.

Despite such a wide range of promising applications, the underlying mechanisms, intrinsic and field-induced interactions of the MMNPs in suspensions are far from being understood. There are several theoretical works elucidating the impact of magnetic interactions (dipole–dipole and/or exchange ones) between grains on equilibrium^49–52^ and magnetodynamic^53–55^ response of a MMNP to an applied field. These works indicated fundamental similarities and profound differences between MMNPs and droplets in ferrofluid emulsions.^56–58^ The similarities stem from the demagnetising effects, while the differences arise due to the spatial constraints. The latter is related to the fact that the MMNP grains are fixed in space and can only move together with the MMNP in contrast to ferroparticles in droplets that can freely diffuse inside. What remains unclear up to now is how the MMNPs self-assemble and what are the main parameters to alter, if one aims to control the clustering in these systems.

In this work, we will employ Langevin dynamics simulations and analytical theory in order to elucidate (i) MMNPs self-assembly in a wide range of magnetic interactions; (ii) the impact of MMNP self-assembly on the static magnetic susceptibility; in thermodynamic equilibrium.

Both questions raised above are particularly interesting as multicore magnetic particles appear to be similar to colloids with multiple patches,^59–63^ but due to the possibility of the granular dipoles to reorient, multicores might exhibit a certain proximity to particles with mobile patches.^64–66^ Moreover, recent experimental studies revealed a difference in self-assembly of magnetic nanoparticles and complex multicore colloids obtained by oil phase evaporation-induced self-assembly of hydrophobic nanoparticles.^67^

The manuscript is organised as follows. Firstly, we analyse the initial susceptibility and find that, depending on the magnetic interactions between the grains, there are two distinct regimes: the system is dominated by demagnitising effects within individual MMNPs and remains very weakly susceptible; the system exhibits a steep increase in the initial susceptibility. Secondly, we show that this steep increase is caused by the MMNPs self-assembly. Within the clusters, MMNPs magnetise each other and become more correlated, albeit not as much as one could expect if the MMNPs were forming chains similar to their single-domain counterparts. Finally, we show that the reason for the grains to not form linear highly correlated structures is the formation of bridges between the MMNPs – clusters of correlated grains that go cross neighbouring MMNPs. Through bridges a MMNP might connect to more than two nearest neighbours. As a result, the clusters formed in the suspensions of MMNPs are more compact than those made by single-domain nanoparticles on the one hand, but, on the other, they are less compact than the bundle-like structures formed by magnetisable non-composite micron-sized magnetic particles.

## 2 Model of a multicore-based ferrofluid

We consider the system of *N* spherical MMNPs with the diameter *D* and volume *v* = π*D*^3^/6, dispersed in a three-dimensional nonmagnetic liquid medium with volume *V*.

Their volume fraction in the dispersion is1
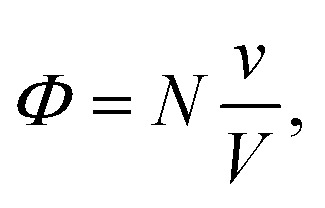


**Fig. 1:**
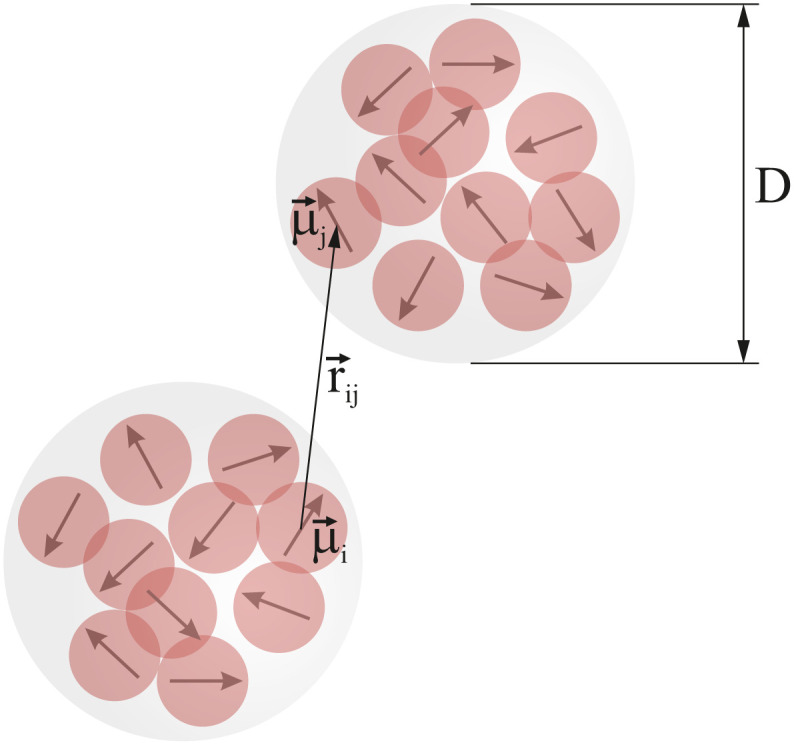
Sketch of the investigated system.

Each MMNP is filled with *n*_g_ spherical magnetic grains of diameter *d* (see Fig. 1). Positions of grains inside MMNPs are rigidly fixed. Grains are placed randomly and uniformly, without overlapping. Volume fraction of grains within each MMNP is2
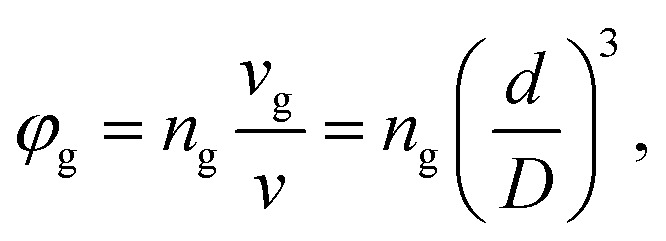
where *v*_g_ = π*d*^3^/6 is the grain volume. MMNPs in the medium are subject to both translational and rotational Brownian motion.

The system is thermostated and has a constant temperature *T*, the external magnetic field is absent.

Grains are assumed to be single-domain. Each grain carries a magnetic moment *μ⃗* of constant magnitude. We will make a simplifying assumption that the internal magnetic anisotropy energy of grains is negligible compared to thermal energy *k*_B_*T* (*k*_B_ is the Boltzmann constant), and that magnetic moments can freely rotate under a combined effect of thermal fluctuations and a total dipolar field created by all other grains in the system. However, it is important to note that zero-field equilibrium properties of immobilized single-domain particles do not depend on the anisotropy energy, if the orientation distribution of their easy axes is random and uniform.^68–70^ Such superparamagnetic systems sometimes referred to as “non-textured”.^71^ Therefore, the results obtained here for isotropic grains can also be extrapolated to non-textured MMNPs with anisotropic grains.

We account for the magnetic *intergrain* interactions by means of the conventional dipole–dipole pair potential3
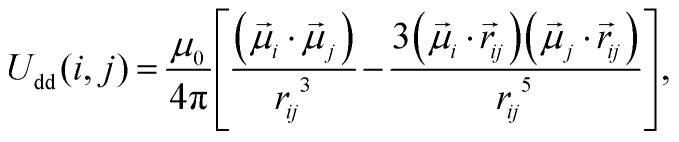
where *μ⃗*_*i*_ and *μ⃗*_*j*_ are the dipole moments of grains *i* and *j*, *r⃑*_*ij*_ = *r⃑*_*i*_ − *r⃑*_*j*_ is the displacement vector connecting their centers and *r*_*ij*_ = |*r⃑*_*ij*_|, *μ*_0_ is the magnetic permeability of vacuum. For characterizing magnetic interactions in our system we use the grain–grain dipolar coupling parameter4
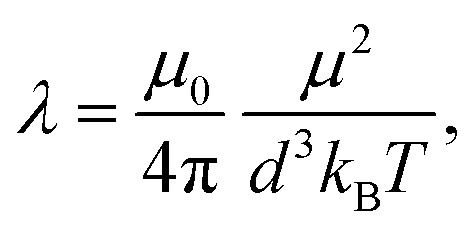
that is the characteristic energy scale of two adjacent grains with dipole moments, divided by *k*_B_*T*.

In experiment, the number of grains per MMNP can vary from tens to hundreds.^48^ In this work we will consider *n*_g_ = 20. This specific value was chosen as a practical compromise. On the one hand, it is big enough so that ensemble-averaged properties of the system can be analysed within a continuum-like approach.^51^ On the other, it is small enough so that we can simulate a suspension within a reasonable time frame. The grain packing fraction within MMNPs will be fixed to *φ*_g_ = 0.2. According to our packing fraction definition, eqn(2), it corresponds to a MMNP diameter of *D* ≃ 4.64*d*. For a typical value of *d* = 10 nm, *D* ≃ 46 nm. Thus, both the number of grains and the nanoparticle linear size lie within experimentally realistic ranges.^72^ It is worth noting, that for such MMNP configuration the majority of grains are actually positioned close to the nanoparticle surface. The fraction of near-surface grains can roughly be estimated as *v*_surf_/*v* = 1 − (1 − 2*d*/*D*)^3^ ≃ 0.8, where *v*_surf_ is the volume of an MMNP outermost spherical shell of thickness *d*. This fact further strengthens the analogy between our system and patchy colloids pointed out in Section 1. An additional discussion on the potential importance of *n*_g_ and *φ*_g_ will be given in Section 3.2.2.

Notice, that in our model the grains are basically close-packed inside the multicore colloid and are not separated by any significant steric layers, similarly to the experimental realisation in ref. 73. In general the presence of the layers between the grains can lead to a decrease of the integrain interactions and as a result affect strongly the magnetic response of such particles to an applied magnetic field, as reported in the experimental study.^74^

The main variables of the work are dipolar coupling constant 0 ≤ *λ* ≤ 10 and MMNP concentration 0.02 ≤ *Φ* ≤ 0.2. Langevin dynamics simulations will be used to determine equilibrium properties of MMNP suspensions within these parameter ranges. All the technical details of the method are given in Appendix A.

## 3 Results and discussion

### 3.1 Initial magnetic susceptibility

The key quantity that characterizes equilibrium zero-field properties of ferrofluids is the initial magnetic susceptibility *χ* (*i.e.*, the initial slope of the magnetization curve). It can be calculated as^75^5
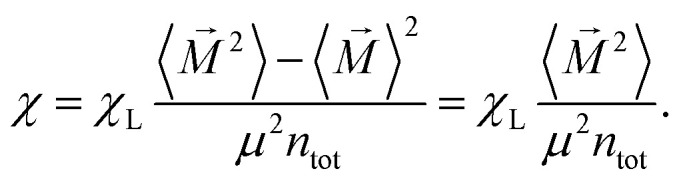
Here, *n*_tot_ = *n*_g_·*N* is the total number of dipoles (grains), *M⃑* is the total magnetic moment of the system,6
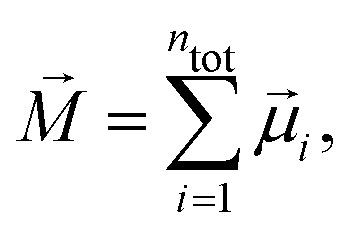
〈⋯〉 denotes an ensemble average, 〈*M⃑*〉 is zero for our system in thermodynamic equilibrium due to symmetry consideration (there are no special directions in our system and all orientations are equally probable), *χ*_L_ is the so-called Langevin susceptibility,7
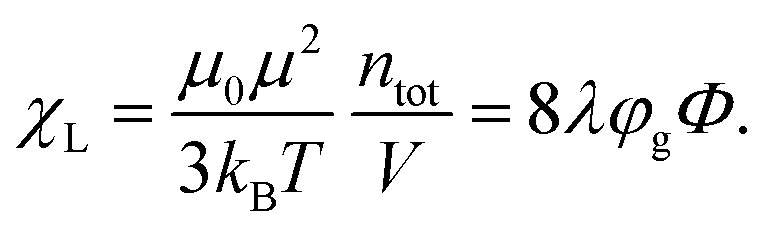
Langevin susceptibility characterizes the initial magnetostatic response of an ensemble of non-interacting dipoles (*i.e.*, of an ideal superparamagnetic gas). Recently, we have suggested two simple analytical estimations for the susceptibility of MMNP ensembles.^52^ The first one takes into account dipole–dipole interactions between grains within individual MMNPs (including demagnetization effect), but neglects interactions between different MMNPs:8
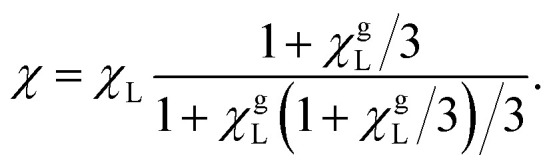
Here, *χ*^g^_L_ is the Langevin susceptibility, corresponding to a MMNP interior,9
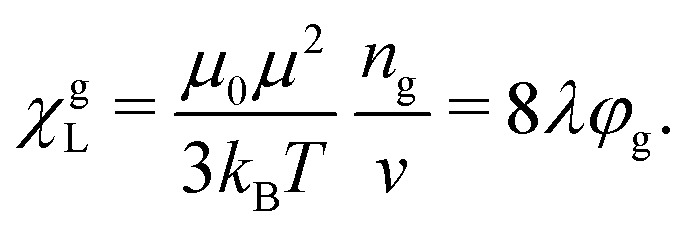
eqn (8) was derived within the so-called modified mean-field theory.^76^ The second equation adds a correction to take into account MMNPs' mutual magnetization (similarly to how it was done for ferroemulsions in ref. 56):10
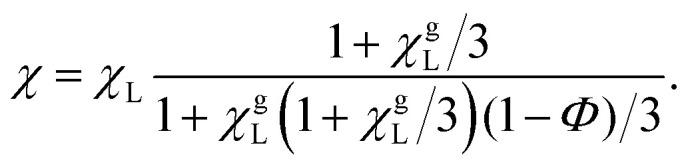


**Fig. 2:**
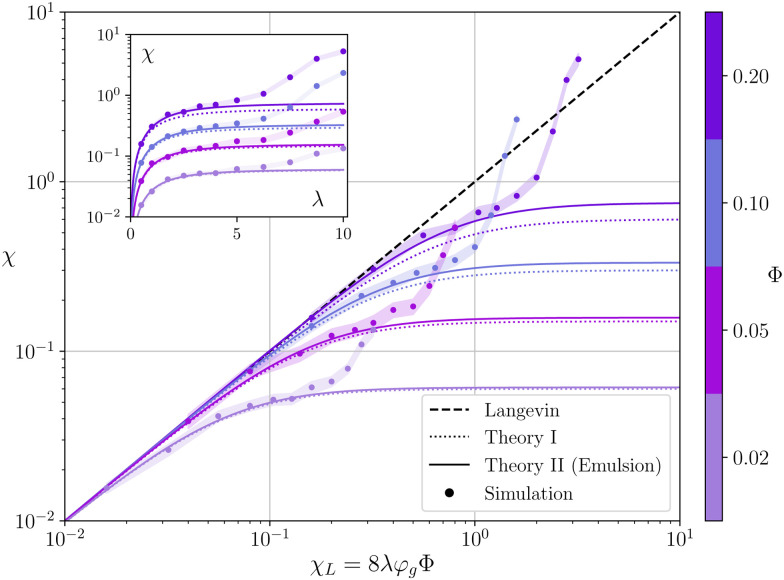
Initial magnetic susceptibility of a multicore-based ferrofluid *vs.* the corresponding Langevin susceptibility *χ*_L_. Different colors correspond to different volume fractions of MMNPs (see colorbar). Circles show simulation results for *n*_g_ = 20 and *φ*_g_ = 0.2 (filled regions indicate 95% confidence intervals), dotted lines – theoretical predictions from eqn (8), Theory I, solid lines – theoretical predictions from eqn (10), Theory II. The dashed line corresponds to the susceptibility of an ideal superparamagnet with *χ* = *χ*_L_. In the inset the same susceptibility values are plotted against the grain–grain dipolar coupling parameter *λ*.

Simulation results for susceptibilities at different ferrofluid concentrations *Φ* are shown in Fig. 2. Values of *χ* are plotted against corresponding Langevin values *χ*_L_ (note the logarithmic scales). Using this representation it is easy to demonstrate the qualitative effect of dipole–dipole interactions on the system. If the susceptibility lies below the diagonal *χ* = *χ*_L_, then the dipoles on average will try to prevent each other from aligning with an applied field, the overall magnetic response of the system will decrease. On the contrary, susceptibility values *χ* > *χ*_L_ indicate a reinforcing role of dipolar interactions. From this standpoint, simulation susceptibility curves in Fig. 2 demonstrate a non-trivial behaviour. In general, the Langevin ideal gas approximation only works in the limit *χ*_L_ ≪ 1 for all investigated samples. After that the susceptibility growth slows down and all the simulation curves fall below the diagonal. This behaviour is well captured by eqn (8) and (10). The predictions from two equations are close, the difference only becomes noticeable for the most concentrated samples. For *Φ* = 0.2, eqn (10) gives 25% larger susceptibilities than eqn (8), and it is actually much closer to simulation data up to *χ*_L_ ∼ 1. It indicates that at least for intermediate *χ*_L_ values the interactions between MMNPs are being taken into account correctly by eqn (10). However, both theories predict the susceptibility saturation at *χ*_L_ ≫ 1, which is mainly due to demagnetization effect within individual MMNPs.^51,52^ Simulation curves, instead of reaching saturation, demonstrate a steep increase. This rapid change is happening at different *χ*_L_ for different samples. The inset in Fig. 2 shows the same susceptibility data plotted against dipolar coupling parameter *λ*. From these plots it is clear that the susceptibility increase is almost exclusively governed by an increase in *λ*. For our systems, it is always happening at *λ* ≳ 6, regardless the concentration value. The last important observation from Fig. 2 is that at *λ* ∼ 10 the susceptibility of concentrated samples with *Φ* ≥ 0.1 can actually reach values above Langevin ones. It means that the overall influence of magnetic interactions can switch from the suppression of the effective field–dipole coupling to its amplification.

We already know that at least in the presence of a strong applied field an anomalous increase in the magnetic response of MMNP ensembles is accompanied by a pronounced self-assembly.^52^ To understand whether the same is true in a zero-field case, a detailed cluster analysis was performed for one of the investigated concentration values, namely for a ferrofluid with *Φ* = 0.02. As the increase in susceptibility is mainly related to the change of *λ*, it is more important to vary the latter, rather than unnecessarily increase the set of sampling parameters by considering multiple concentrations and multiple magnetic coupling parameters at the same time.

### 3.2 Microstructure of a multicore-based ferrofluid

**Fig. 3:**
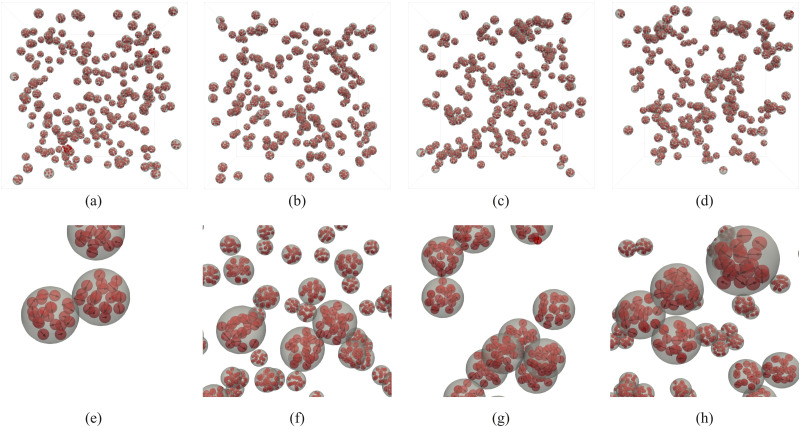
Representative simulation snapshots of the investigated systems for various values of dipole–dipole interactions: (a) and (e) *λ* = 6.25; (b) and (f) *λ* = 7.5; (c) and (g) *λ* = 8.75, (d) and (h) *λ* = 10. The bottom row is zoom in the clusters found in the dispersions from the upper row.

Looking at the upper row in Fig. 3, where the characteristic snapshots of the MMNP systems are presented, one can see how from an almost uniformly distributed in space (Fig. 3(a)) the dispersion exhibits a clear structural transformation if the value of *λ* increases and quite large clusters of MMNP are formed (Fig. 3(d)). Here, subfigures (a) and (e) are for *λ* = 6.25; (b) and (f) for *λ* = 7.5; (c) and (g) for *λ* = 8.75; and (d) and (h) for *λ* = 10. Even at the first glance one can notice that the topology of the clusters in Fig. 3 is notably different from those formed by dipolar hard or soft spheres. Compare, for example snapshots from works^77,78^ to those in Fig. 3. Single-core magnetic particles mainly form structures with long linear segments and only few junctions. MMNPs, instead, seem to form more compact aggregates. If we zoom in the clusters, see the lower row of Fig. 3(e)–(h), some alignment of the dipoles in the granules of adjacent MNNPs can be observed. Thus, it is clear that the clustering in such system is occurring on two different spatial resolutions: MNNPs and individual granules. Below, as a consequence, we consider two different length-scales in order to explain equilibrium self-assembly of MMNPs.

First, assuming each MMNP centre of mass to be a vertex of a graph, we identify the graph of connected components, meaning the clusters of MMNPs. The choice of a connected component is based on the definition of the edge connecting the two vertices in the graph. We consider two MMNPs as connected by an edge if the distance between their centres does not exceed 2^1/6^*D*, and the total magnetic interaction between the two MMNPs is attractive, *U*_dd_ ≤ 0.

Second, we increase the resolution and look at the arrangement of individual grains in the previously defined clusters. We analyse how the grains of two adjacent MMNPs orient their magnetic dipoles in order to form the bond.

#### 3.2.1 Clusters of MMNPs

**Fig. 4:**
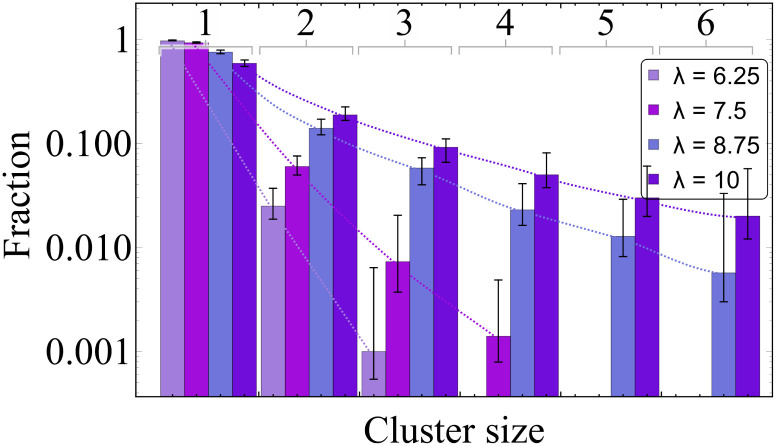
Cluster size distribution of a multicore-based ferrofluid. Values of dipole–dipole interactions are given in the legend. Dotted lines are guide for an eye to appreciate the deviations from the exponential decay observed for high values of *λ*.

In Fig. 4 we plot the cluster-size distributions. The vertical axis shows the probability of finding a cluster of a size shown along the horizontal axis. Each colour corresponds to a certain value of the magnetic coupling, as shown in the legend. This probability in case of single-core dipolar hard or soft spheres has an exponential form and should be linear if a log-scale is applied along the vertical axis as it is done in Fig. 4. However, as fits plotted with dotted lines show, only the decay *λ* = 6.25 might have been exponential (the third point should be taken with caution as there are very few clusters of the size three), all others are not. One can see that for the smallest value of *λ*, only about one per cent of dimers are there in the systems and the probability of finding a three particle cluster is negligible. For *λ* = 10, instead, only 60 per cent of MNNPs remain non-aggregated, while the rest of the system forms relatively large clusters.

**Fig. 5:**
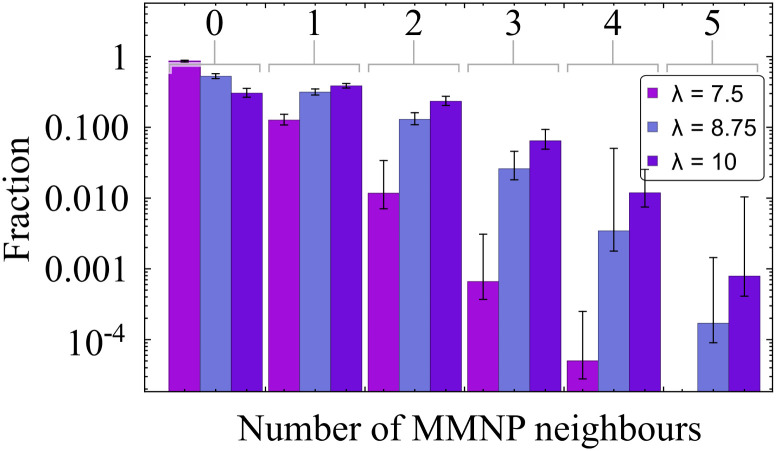
Fraction of MMNPs having 0, 1, 2, 3, 4 or 5 neighbours. Values of dipole–dipole interactions are given in the legend.

As previously mentioned, not only the cluster size distributions, but also the topology of clusters formed by MMNPs seems to deviate from that of the dipolar hard or soft sphere particles. In order to verify this, for each MMNP, we calculate the number of nearest neighbours, *i.e.* the degree of each graph vertex. The histogram of MMNP degrees is shown in Fig. 5.

Here, the same colour code as in Fig. 4 is used, although the results for *λ* = 6.25 are not provided as the majority of MMNPs are non-clustered. For *λ* = 7.5, only one per cent of MMNPs has two and more neighbours, meaning that only dimers form. For *λ* = 10, instead, more than 35 per cent of MMNPs have two and more neighbours, 30 per cent are having no neighbours. The remaining 35 per cent have one neighbour. The latter value shows that the number of open ends (vertices with degree one) typical for chain-like structures, or linear segments of branched clusters increases with magnetic interaction energy.

**Fig. 6:**
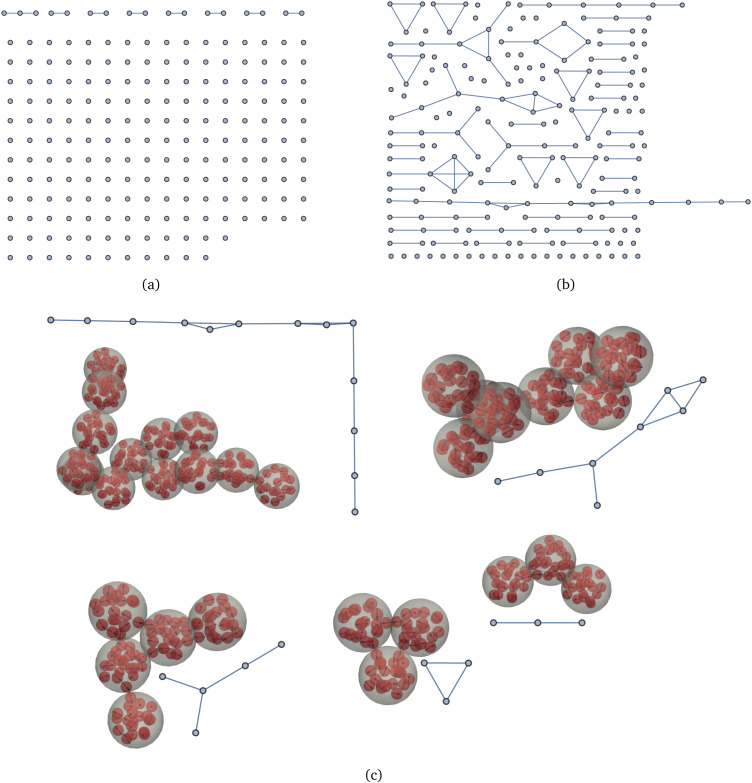
Typical graphs of the investigated MMNP dispersions for various values of dipole–dipole interactions: (a) *λ* = 6.25; (b) *λ* = 10. (c) Correspondence between graph representations and real clusters for *λ* = 10.

Even clearer picture of the cluster distribution and their topology can be obtained looking at Fig. 6. Here, typical graph representations of two simulation snapshots in equilibrium are presented. If *λ* = 6.25, Fig. 6(a), we observe one trimer, and seven dimers, all other MMNPs are nonaggregated. The system looks very different for *λ* = 10, Fig. 6(b). Here, we find several big connected components with vertices in them with degree two and higher. One should not be confused by stretched systems, as the representation of the graphs is simply optimised to occupy as few space as possible. In reality, those large clusters are 3D and rather compact, as the snapshot in Fig. 6(c) indicates. Here, for *λ* = 10, we show representative 3D structures of large clusters together with their graph representations, in order to avoid being confused by seemingly linear graphs.

Previously, it was shown that the chain formation in single-core systems leads to the increase of the system magnetic response,^79^ while the formation of rings reduces the susceptibility.^80^ Below, we investigate how the new cluster topologies manifest themselves in the MMNP suspension magnetic properties.

#### 3.2.2 Contribution of clusters into magnetostatic response

Combining the knowledge obtained form the inset of Fig. 2 and 4 it is obvious that the clustering of MMNPs and a susceptibility steep increase are happening at *λ* ≳ 6, so they must be correlated. To quantify the connection between two phenomena the following procedure was employed. Using the cluster analysis results, we were able to divide all MMNPs into two subgroups at each simulation step. The first subgroup contains non-clustered (single) MMNPs, let us denote their number as *N*_s_. The second subgroup contains all MMNPs with non-zero degree and their number is denoted by *N*_c_. Considering that the suspension is in a dynamic equilibrium, the values of *N*_c_ and *N*_s_ will fluctuate in time, but the sum of two numbers is fixed (*N*_s_ + *N*_c_ = *N*). We denote the total magnetic moment of all single MMNPs as *M⃑*_s_, and the total magnetic moment of all clustered MMNPs as *M⃑*_c_. Evidently, the total magnetisation of the suspension is the sum of the two, *M⃑* = *M⃑*_s_ + *M⃑*_c_. Using these definitions, we can rewrite the initial susceptibility eqn (5) as

11 *χ* = *χ*_s_ + *χ*_c_ + Δ*χ*.

Here, the expressions for the subgroups are:12
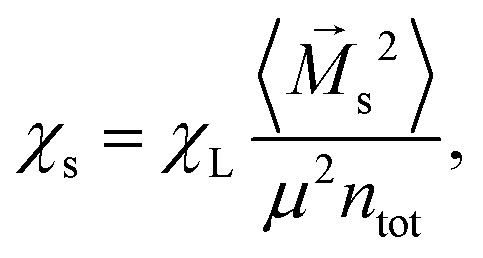
13
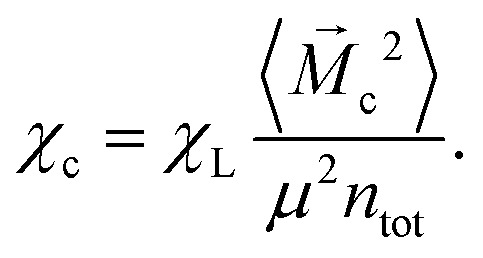
The last term in eqn (11) takes into account correlations between single particles and clusters:14
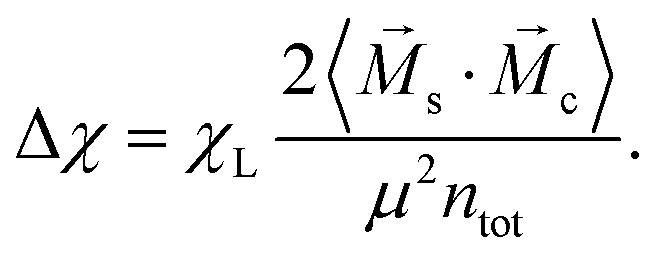
Of course, from the very nature of single particles, one can reasonably expect Δ*χ* to be small.

**Fig. 7:**
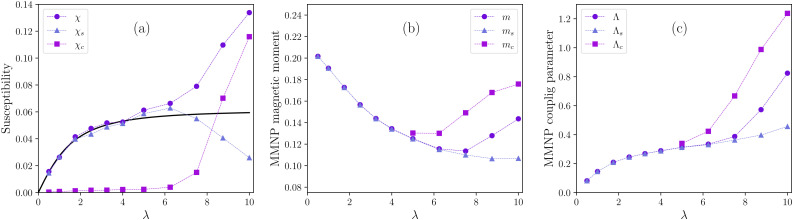
Zero-field magnetic characteristics of a multicore-based ferrofluid *versus* grain–grain dipolar coupling parameter *λ*. *Φ* = 0.02. Circles are simulation results averaged over the whole system, triangles correspond only to non-clustered (soliraty) MMNPs and squares correspond to clustered MMNPs. (a) Initial magnetic susceptibility, solid line is from eqn (10); (b) average normalized magnetic moment of a MMNP; (c) effective parameter of dipolar coupling between a pair of MMNPs.

Fig. 7(a) shows how *χ*_s_ and *χ*_c_ change with growing *λ* for *Φ* = 0.02. For reference, in the same figure we also provide the total susceptibility *χ* – both numerical and analytical – repeating the data from the inset of Fig. 2. Values of the correlation term are of the order of |Δ*χ*| ∼ 10^−3^–10^−2^ and are not shown. It is seen that up to the point where eqn (10) is still working, the susceptibility is mostly determined by single MMNPs, while the contribution of clusters is negligible (as well as their fraction in the system). After that, as the dipolar coupling parameter *λ* increases from 6.25 to 10, *χ*_s_ drops approximately by the factor of two and a half, that agrees well with Fig. 4, where the actual number of single particles drops from almost 200 at *λ* = 6.25 to approximately 80 at *λ* = 10.

In contrast to the susceptibility of single particles, the value of *χ*_c_ grows rapidly and at *λ* = 10 is twice as high as the theoretical saturation value *χ* ≃ 0.061 (as predicted by eqn (10)).

Another quantity that, along with susceptibility, can provide insights into magnetic properties of our system is the average magnetic moment of a MMNP. We calculate it as15
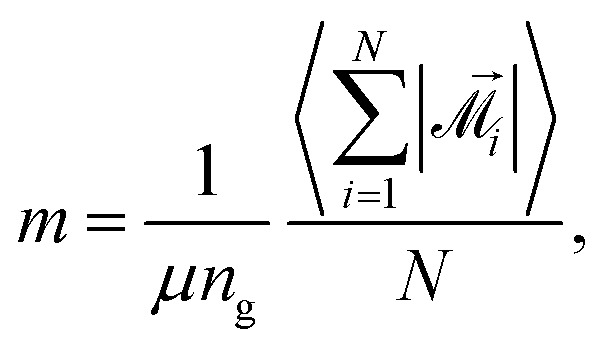
where 
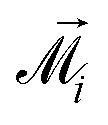
 is the total instantaneous magnetic moment of the *i*th MMNP,16
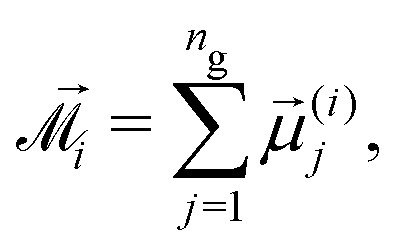
Vectors *μ⃗*^(*i*)^_*j*_ are the magnetic moments of the grains in the *i*th MMNP. The average magnetic moment *m* is normalized, its maximal value *m* = 1 corresponds to the magnetic saturation. If interactions between magnetic moments of grains are weak, *m* must be close to *n*_g_^−1/2^ ≃ 0.22. We can also calculate average magnetic moments separately for two particle subgroups:17
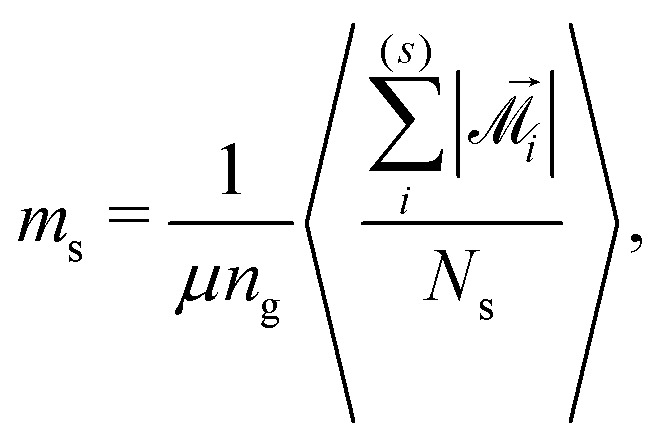
18
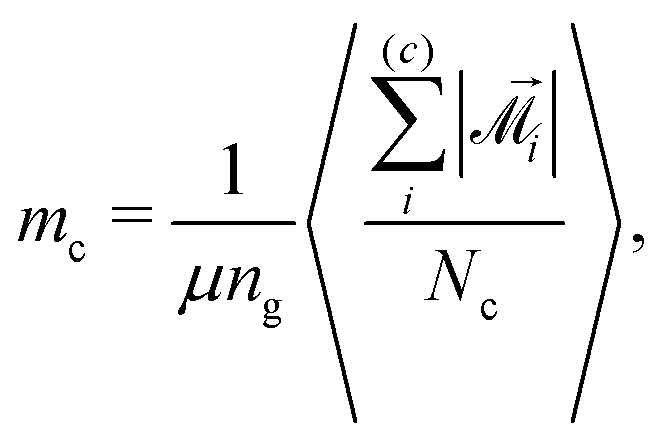
where 
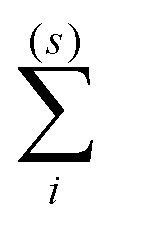
 and 
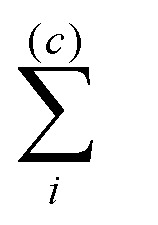
 denote summation over single and clustered MMNPs, respectively.

In Fig. 7(b) we plot dependencies of magnetic moments *m*, *m*_s_ and *m*_c_ on *λ*. Considering that the MMNPs form a statistically relevant number of clusters only for *λ* ≥ 5, at lower coupling constants the number of clustered particles *N*_c_ is too small to reliably estimate *m*_c_ from eqn (18). Initially, magnetic moments are decreasing with *λ*. This is the result of the demagnetisation effect – for a localised collection of interacting dipoles surrounded by a non-magnetic medium it is always energetically more favorable to minimise its total magnetic moment.^81^ However, while for single particles this decrease continues up to *λ* = 10, the dependence of *m* is found to be non-monotonic. At *λ* ≳ 6, as the fraction and the average size of clusters start to grow, the magnetic moment of clustered particles turns into an increasing function as well. MMNPs within clusters, therefore, help each other to overcome the demagnetisation effect and become more susceptible to the applied field.

Finally, knowing numerical values for MMNPs magnetic moments, we can draw a comparison with dipolar spheres, which are used for modelling of traditional single-core-based ferrofluids. For each value of *λ*, instead of an ensemble of MMNPs, let us consider an equivalent ensemble of dipolar spheres of diameter *D* with a point-like dipole in its center. The magnitude of this magnetic moment is constant and equals to 
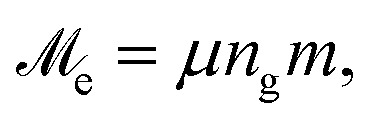
 where *m* is calculated from eqn (15). Then similarly to eqn (4) we can introduce an effective dipolar coupling parameter for a pair of MMNPs as19
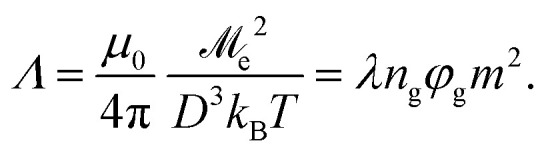
A notable thing in eqn (19) is that it suggests that interactions between MMNPs must strongly depend on both number of grains *n*_g_ and their packing fraction *φ*_g_. We expect that changing these parameters can change a critical value of *λ*, at which the self-assembly starts to influence system magnetic response (in our case, this is *λ* ≃ 6). A detailed inquiry into the importance of *n*_g_ and *φ*_g_ is left for future studies.

Values of *Λ* for *n*_g_ = 20 and *φ*_g_ = 0.02 are shown in Fig. 7(c). We can see that it actually remains quite low, *Λ* < 1. If we estimate the coupling parameter independently for two particle subgroups (*Λ*_s_ = *λn*_g_*φ*_g_*m*_s_^2^ and *Λ*_c_ = *λn*_g_*φ*_g_*m*_c_^2^), it will be somewhat larger for clustered particles and can reach *Λ*_c_ = 1.2. But we know that for real dipolar spheres a pronounced self-assembly in the absence of a field only takes place at *Λ* ≥ 4^77,82^! Therefore, the crude analogy with dipolar spheres is insufficient to explain the self-assembly mechanism in multicore-based ferrofluids. An obvious difference between two systems is that the total magnetic moment of a multicore particle is not point-like, it is distributed across the particle volume. Presumably, correlations between individual grains within interacting MMNPs must be explicitly taken into account in order to explain the observed behavior. In fact, below we reveal the mechanism that keeps MMNPs together even though the effective *Λ* between them is very low.

#### 3.2.3 Bridges between multicore nanoparticles

**Fig. 8:**
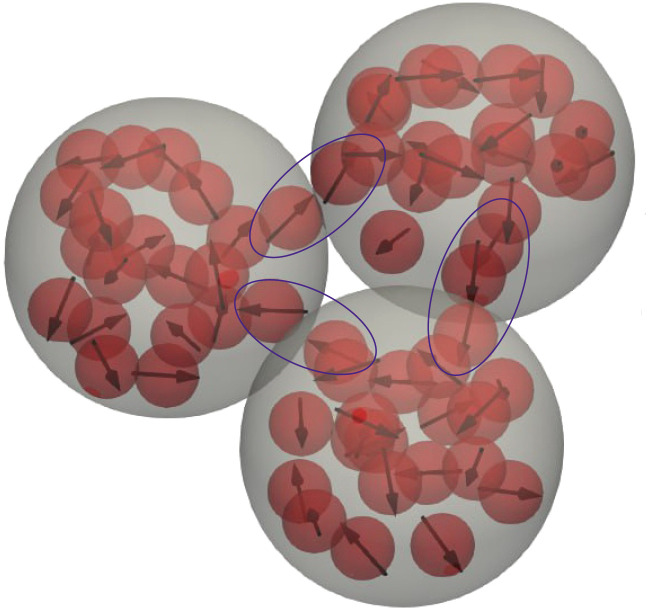
Snapshot of a MMNP cluster. Grains on the borders between different MMNPs, forming bridges, are encircled.

Increasing the resolution of the analysis, in this subsection, we study the internal structure of clusters, namely, the correlations between the grains inside MMNPs. Looking closer at Fig. 3(e)–(h), one can notice that individual grains from different MMNPs cooperate and form their own internal clusters. Such type of clusters, consisting of grains belonging to different MMNPs, we call bridges and provide a more detailed snapshot in Fig. 8.

In order to analyze the structure and number of bridges, we once again carried out a cluster analysis. Firstly, we localize all MMNP clusters as connected components (see, explanations above) and sort them into groups according to the number of MMNPs in them. Secondly, for each such element of each subgroup, we construct a graph whose vertices are the individual grains and find connected components within these new structures using two conditions (i) two grains are connected if the distance between them is smaller than 2^1/6^*d* and the dipolar interaction between them is negative; (ii) the connected component is a cluster of interest if it contains vertices from different MMNPs. Finally, any property of bridges that is calculated is averaged over the MMNP clusters of a given size.

**Fig. 9:**
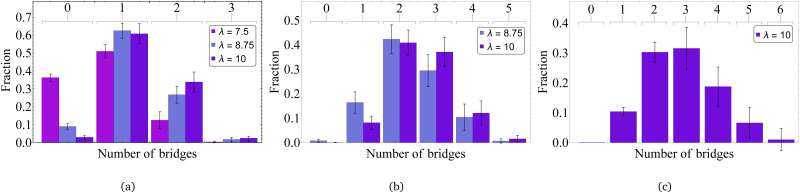
Number of bridges in MMNP clusters of different sizes: (a) cluster size equals 2, (b) cluster size equals 3, (c) cluster size equals 4. Values of dipole–dipole interactions are given in the legend.

In Fig. 9, we present the probability of finding a certain number of bridges within the cluster of two (a), three (b) and four (c) MMNPs. If *λ* = 7.5 the only reliable statistics can be obtained for dimers. Here, half of the clusters have at least one bridge, approximately 12 per cent have two bridges holding the particles together. We believe, that 35 per cent of clusters without bridges is related to a very restrictive distance criteria that we apply to detect clusters. In fact, with growing dipolar coupling one sees that the number of dimers without connecting bridges vanishes, while the probability of finding two bridges is increasing. Three particle clusters are normally connected with two-three bridges. Four-particle clusters are forming frequently enough only for *λ* = 10. Here within the 10 per cent margin one can find up to five bridges.

**Fig. 10:**
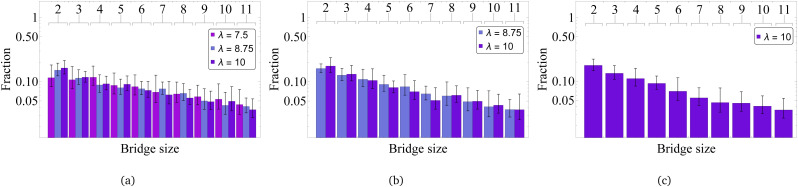
Bridge size in MMNP clusters of different values: (a) cluster size equals 2, (b) cluster size equals 3, (c) cluster size equals 4. Values of dipole–dipole interactions are given in the legend.

In Fig. 10 one can see that the bridges are rather long. With only slightly decreasing probability, one can find bridges with length from two to seven particles in dimers Fig. 10(a). Interestingly enough, comparing Fig. 10(b) and (c), where the bridge length distributions are plotted for three- and four-particle clusters respectively, to the histogram for dimers in (a), one concludes that the difference is barely to be found. In other words, the length of the bridges is cluster-size independent and it is only weakly affected by *λ*. This suggests that the bridges that we find reach their limiting length determined by spatial constraints of the MMNP size. In fact, if grains follow MMNP diameter, only 4 of them can fit inside. Taking this into account, it is particularly curious to elucidate what is a typical bridge topology and if it depends on the bridge length.

**Fig. 11:**
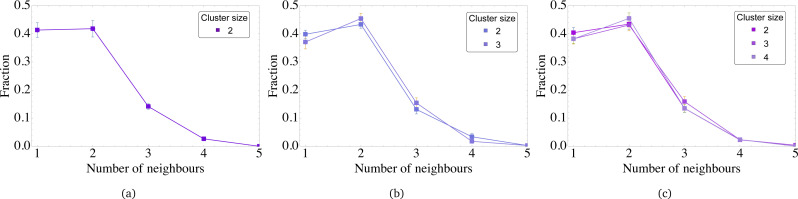
Fraction of grains having a given number of neighbours in bridges for various dipole–dipole interactions: (a) *λ* = 7.5, (b) *λ* = 8.75, (c) *λ* = 10. The averaging is performed among MMNP clusters of different sizes as shown in the legend.

The first step to define the topology of the bridges is to calculate the degree of the grains in bridges. The results are collected in Fig. 11. Had the bridges been chains, there would have been only grains with degrees one and two. If, for example, the chain-like bridge was built of six grains, roughly 33 per cent would have degree one and 67 per cent degree two. The longer the chain, the lower the fraction of free ends in comparison to the grains with degree two. Here, however independently from the values of *λ* and the cluster size, 35–40 per cent of grains have degree one, 40–45 per cent degree two, around 15 per cent of grains in bridges have degree of three and five per cent of grains have four different neighbours.

**Fig. 12:**
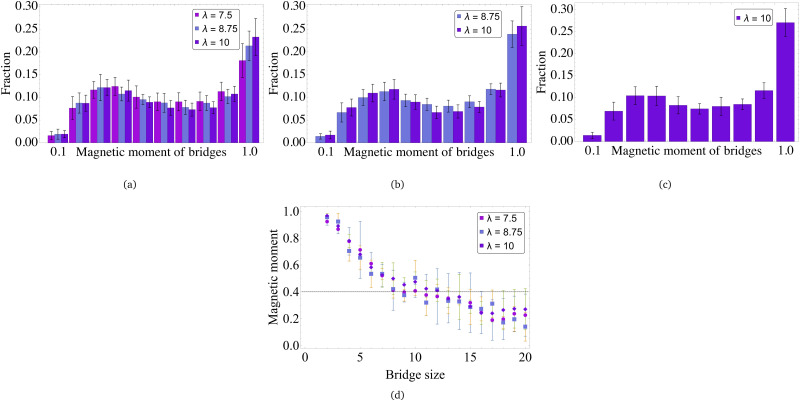
Average magnetic moment of bridges in MMNP clusters of different sizes: (a) cluster size equals 2, (b) cluster size equals 3, (c) cluster size equals 4. (d) The magnetic moment of bridges depending on the bridge size. Values of dipole–dipole interactions are given in the legend.

Fig. 12(a)–(c) show the total magnetic moment of the bridge, normalized by the magnitude of the grain magnetic moment and bridge length. It can be seen that for all values of *λ* and cluster sizes, there is a bimodal distribution of the magnetic moment: the first peak in the region of 0.2–0.3 and there is a main maximum near unity. The latter corresponds to the linear structure of the bridge, whereas the peak at 0.2 indicates some bend, possibly branched or closed, configuration. Importantly, the bend structures are not ideal rings, as the magnetisation is not that low as one would expect for a mildly perturbed ring. Fig. 12(d) shows the dependence of the average bridge magnetic moment on the bridge length. It confirms the presence of long banded or nearly closed structures: short bridges have a very high magnetic moment, while the second histogram maximum in Fig. 12(a)–(c), shown by a vertical line, corresponds to the bridges longer than 10. It means that grains with degrees three and four are the bending points. Long banded bridges often start and end in the same MMNP, while the part in the fold belongs to the neighbouring MMNP in the cluster. Only few very long non-banded bridges were found in simulations that would percolate through three or more grains.

The formation of various bridges allows to increase the number of nearest neighbours a MMNP can have, making it similar to colloids with mobile multiple patches.^83^

## 4 Conclusions

In this work, using Langevin dynamics computer simulations accompanied by analytical calculations, we investigated diluted suspensions of multicore magnetic nanosized particles (MMNPs) composed by grains, whose positions are fixed within the particle body, but their magnetic moments are free to rotate, corresponding to the grains with negligibly low magnetic anisotropy.

Self-assembly of MMNPs and its impact on the zero-field magnetostatic response of the MMNPs suspensions were addressed at two different scales.

Firstly, it was shown that MMNPs started forming clusters at the values of saturation magnetisation much higher than their point-dipolar counterparts. Instead of forming inherent to dipolar spheres chains or rings whose size distribution decays exponentially with the cluster size, for higher interaction strength, the clusters formed by MMNPs are more compact, and their sizes are not distributed exponentially. In fact, the total average magnetic moment of a particle is rather small, and for the conditions in which self-assembly does not take place, the static susceptibility of MMNP suspensions is found to be below Langevin one and to increase only slightly with growing magnetic coupling parameter. At the value of *λ* at which the self-assembly ushers in, the susceptibility of the system qualitatively changes, starting growing very fast and going above the susceptibility of an ideal superparamagnetic gas predicted by Langevin theory.

Secondly, in order to understand the structure of the clusters formed by MMNPs, we looked at the interactions of the individual grains in neighbouring MMNPs. We discovered that clustering occurs by means of building grain bridges: grains of kissing MMNPs reorient so that they can form clusters. The length of those clusters (bridges) is found to be only weakly dependent on the magnetic interaction strength if the latter is high enough. One can find bridges whose length varies between 3–4 grains to 7–11 of them. Short bridges are mainly linear and have a relatively large magnetic moment. Long bridges instead have banded, u-like shape that is nearly closed with rather small total magnetic moment compensated by their folded structure.

Bridge formations is responsible for the multiple bonds that MMNPs can form, attaching more than two nearest neighbours.

This study reveals a mechanism of MMNPs self-assembly that makes this system clearly different from both single-domain nanoparticles and magnetisable micron-sized colloids. As the next step, we would address the dynamics of MMNPs.

Even though, the direct discussion of the dynamical properties of multicore suspensions cannot be based on the results presented in this paper, we cannot but mention the potential impact of structural transitions on the dynamic susceptibility of these systems. We assume that the formation of bridges as a mean for multicore particles to self-assemble will lead to a significant broadening of the dynamic spectra. At the moment we are working on the generalisation of our approach to capture off-equilibrium dynamics.

## Conflicts of interest

There are no conflicts to declare.

## Supplementary Material

## Appendices

### A simulation details

All the results reported here are from molecular dynamics simulations performed in the software package ESPResSo 4.1.4.^84^ The “virtual sites” feature of ESPResSo is used to take into account the granular nature of MMNPs.^85^ Each magnetic grain is treated as a virtual site, connected to the center of the corresponding “host” MMNP. The position of each virtual site is rigidly fixed within the body reference frame of the host. All forces and torques acting on virtual sites are propagated back to the host on every simulation time step. Langevin thermostat technique is used to achieve thermodynamic equilibrium. Formally, Langevin equations for translation and rotational motion of a given MMNP can be written as

20

21

where asterisk denotes reduced quantities, *d* is used as a unit of length, grain mass *p* is used as a unit of mass and *k*_B_*T* is used as a unit of energy. Thus,  and  are the reduced linear and angular velocities of a MMNP, respectively, *P** = *P*/*p* is the reduced MMNP mass, *J** = *J*/*pd*^2^ is its reduced moment of inertia,  is the total reduced force acting on a given MMNP, *f⃑*^C^ is the central force acting on a MMNP due to steric interactions, *f⃑*^g^_*j*_ is the total force acting on the *j*th virtual site within MMNP, , *r⃑*^C^_*j*_ is the virtual site position relative to the MMNP center of mass, *τ⃗*^g^_*j*_ is the total torque on the *j*th virtual site,  and  are the reduced translational and rotational friction coefficients,  and  are the random thermal force and torque on a MMNP, respectively. They have zero mean values and are connected to  and *via* standard fluctuation-dissipation relationships.^86^ The reduced time is .

While the “virtual sites” feature is used to calculate the positions of superparamagnetic grains, the equilibrium orientations of their magnetic moments are determined independently from Langevin equations identical in form to eqn (21):

22

Here,  is the reduced magnetic torque acting on a given grain, *H⃑*_dd_ is the sum all dipolar fields in the grain centre, dimensionless quantities , ,  and  have the same meaning as their counterparts from eqn (21).

To simulate the steric repulsion between MMNPs, the Weeks–Chandler–Andersen (WCA) pair potential is used:^87^

23

where *R* is the distance between centers of two MMNPs. Additionally, a similar potential is imposed between each pair of grains in the system:

24

Introduction of this potential was required to avoid numerical instabilities arising in rare cases when the dipole–dipole interaction between two grains from two different MMNPs was large enough to overcome a soft core repulsion modelled by eqn (23) (at *λ* ∼ 10). This approach allowed us to use a larger time step.

All simulations are performed for *N* = 200 MMNPs placed inside a cubic box with a side length of . 3D periodic boundary conditions are imposed on a box. The torques due to long-range dipole–dipole interactions are computed using the dipolar P^3^M algorithm.^88^ Other simulation parameters are *P** = *N*_g_ = 20, *J** = 0.1*P**(*D*/*d*)^2^ ≃ 43, . The simulation time step is Δ*t** = 0.002. Typically, the first 5 × 10^5^ time steps are used for the system equilibration, and the subsequent production run lasts for at least another 10 × 10^5^ time steps. For every combination of *Φ* and *λ* at least five independent runs are performed.

Albeit high efficiency, the choice of such dimensionless parameters imposes a certain “limitation”: the dynamics of our system is not realistic, and we cannot extract any off-equilibrium properties of the system. Although very interesting, the life-time of the clusters and dynamic response of the multicores lay beyond the scope of this study and require more frequent sampling with a smaller time-step, not compatible with reaching equilibrium in a feasible time.
